# Applying MRI Intensity Normalization on Non-Bone Tissues to Facilitate Pseudo-CT Synthesis from MRI

**DOI:** 10.3390/diagnostics11050816

**Published:** 2021-04-30

**Authors:** Kuei-Yuan Hou, Hao-Yuan Lu, Ching-Ching Yang

**Affiliations:** 1 Department of Radiology, Cathay General Hospital, Taipei 106, Taiwan; circle74@gmail.com; 2 Department of Biomedical Imaging and Radiological Sciences, National Yang-Ming Chiao-Tung University, Taipei 711, Taiwan; 3 Institute of Radiological Sciences, Tzu-Chi University of Science and Technology, Hualien 970, Taiwan; s10725007@ems.tcust.edu.tw; 4 Department of Medical Imaging and Radiological Sciences, Kaohsiung Medical University, Kaohsiung 807, Taiwan; 5 Department of Medical Research, Kaohsiung Medical University Chung-Ho Memorial Hospital, Kaohsiung 807, Taiwan

**Keywords:** MRI intensity normalization, pseudo-CT synthesis, convolutional neural network

## Abstract

This study aimed to facilitate pseudo-CT synthesis from MRI by normalizing MRI intensity of the same tissue type to a similar intensity level. MRI intensity normalization was conducted through dividing MRI by a shading map, which is a smoothed ratio image between MRI and a three-intensity mask. Regarding pseudo-CT synthesis from MRI, a conversion model based on a three-layer convolutional neural network was trained and validated. Before MRI intensity normalization, the mean value ± standard deviation of fat tissue in 0.35 T chest MRI was 297 ± 73 (coefficient of variation (CV) = 24.58%), which was 533 ± 91 (CV = 17.07%) in 1.5 T abdominal MRI. The corresponding results were 149 ± 32 (CV = 21.48%) and 148 ± 28 (CV = 18.92%) after intensity normalization. With regards to pseudo-CT synthesis from MRI, the differences in mean values between pseudo-CT and real CT were 3, 15, and 12 HU for soft tissue, fat, and lung/air in 0.35 T chest imaging, respectively, while the corresponding results were 3, 14, and 15 HU in 1.5 T abdominal imaging. Overall, the proposed workflow is reliable in pseudo-CT synthesis from MRI and is more practicable in clinical routine practice compared with deep learning methods, which demand a high level of resources for building a conversion model.

## 1. Introduction

The integration of MRI into radiotherapy is an important technological development to improve tumor targeting [1,2]. MRI provides superior soft tissue contrast to CT, which enables better target delineation, but MRI data do not contain electron density information, which is necessary for accurate dose calculation. Hence, for the MR-LINAC installed in our department, the anatomical information from the daily MRI scan and the electron density information from the planning CT scan are integrated through MRI-CT registration to realize radiotherapy plan adaptation [3,4]. However, image registration in the chest and abdomen is more complex than in the brain because of breath motion, internal organ distortion, and stomach/bladder filling [5]. As the clinical reliability of image registration is not always guaranteed, the introduced error would compromise the effectiveness of treatment and the patient’s quality of life. One solution to the problem is to synthesize pseudo-CT from in-room MRI by using voxel-based methods, which has been considered in attenuation correction of PET/MR imaging [6,7]. Several voxel-based methods have been proposed for pseudo-CT synthesis from MRI, such as the bulk-density method, which assigns homogeneous CT numbers to volumes of interest (VOIs) defined on MRI, and learning-based methods, which employ model fitting or statistical learning techniques [8,9,10,11]. Before applying these voxel-based methods, intensity inhomogeneity in MRI, which arises from the imperfections in the image acquisition process, has to be compensated to provide accurate electron density estimation [12,13,14]. Since inhomogeneity correction methods implemented on different scanners are vendor-specific, different intensities for the same tissue type are observed among different MRI systems even if comparable pulse sequences are used. Consequently, the pseudo-CT synthesis method designed for one MRI scanner cannot be used on other different systems. In our opinion, if these pseudo-CT synthesis methods are generalized across different scanners, they could be more easily embedded within routine practice for either MR-LINAC adaptive plans or PET/MR attenuation correction. The image intensity in MRI is a relative and not an absolute value, which is different from the CT numbers, which represent linear attenuation coefficients calibrated with reference to water. It was hypothesized that the pseudo-CT synthesis method would increase generalization if MR images were normalized to a similar intensity level within the same tissue type. Therefore, the aim of this study was to propose an MRI intensity normalization method to facilitate pseudo-CT synthesis from MRI.

## 2. Materials and Methods

### 2.1. Chest Imaging Acquisition for Cancer Treatment

CT simulation scans for treatment planning were taken on a 16-slice Philips Brilliance Big Bore CT simulator (Philips Healthcare Systems, Andover, MA, USA) with 16 × 1.5 mm collimation and helix scan mode (pitch = 1). The routine protocol for chest scan uses a fixed tube current-time product of 250 mAs at 120 kVp, which results in a CTDI_vol_ of 17.6 mGy. Acquired data were reconstructed using a 512 × 512 matrix with standard FBP algorithm. The voxel size of the reconstructed CT image was 0.98 mm in the axial plane and 3 mm in the longitudinal direction.

The MR-LINAC used in this study (ViewRay MRIdian Linac, ViewRay Inc., Oakwood Village, OH, USA) houses a 0.35 T MRI system, which utilizes a whole-body RF transmit coil and surface receive coils anterior and posterior to the patient. The receive coils consist of radiolucent phased arrays with 2 × 6 channels for the torso. The pulse sequence used for volumetric MRI imaging was a True Fast Imaging with Steady State Precession (TRUFI) sequence, which is a type of balanced steady-state free precession (bSSFP) sequence, yielding a T1-weighted contrast. In-plane field of view (FOV) was 50 cm × 45 cm to cover the whole body. The voxel size of the reconstructed MRI image was 1.5 mm in the axial plane and 3 mm in the longitudinal direction.

### 2.2. Abdominal Imaging Acquisition for Lesion Diagnosis

Diagnostic CT scans were performed on a 64-detector row CT system (Brilliance 64, Philips Medical Systems, Cleveland, OH) using 0.5 s of gantry rotation time. The routine protocol for abdominal scan uses tube current modulation (TCM) at 120 kVp with 5 mm slice thickness. The reference parameter of the TCM system is the dose right index (DRI), which is set as 15 in our routine practice. The acquired data were reconstructed by iDose iterative reconstruction algorithm with standard reconstruction kernel (B). The voxel size of the reconstructed CT image was 0.68 mm in the axial plane and 5 mm in the longitudinal direction.

Diagnostic MRI scans were performed on a 1.5 T system (GE Optima MR450w, GE Medical Systems Inc., Waukesha, WI, USA), which has a flat indexed tabletop to which a constructed receiver coil frame can be attached without patient body contact. The T1-weighted in-phase and out-phase MR image sets were obtained with a 2D fast RF-spoiled dual gradient echo sequence. Echo time was 4.4/2.2 ms, whereas repetition time and flip angle were 110 ms and 80°, respectively. The bandwidth for the entire FOV (34 cm × 34 cm) was 62.5 kHz. The frequency encoding direction was anterior–posterior. Acquired data were reconstructed axially using a 512 × 512 matrix. The voxel size of the reconstructed MRI image was 0.66 mm in the axial plane and 8 mm in the longitudinal direction.

### 2.3. MRI Intensity Normalization

**Figure 1:**
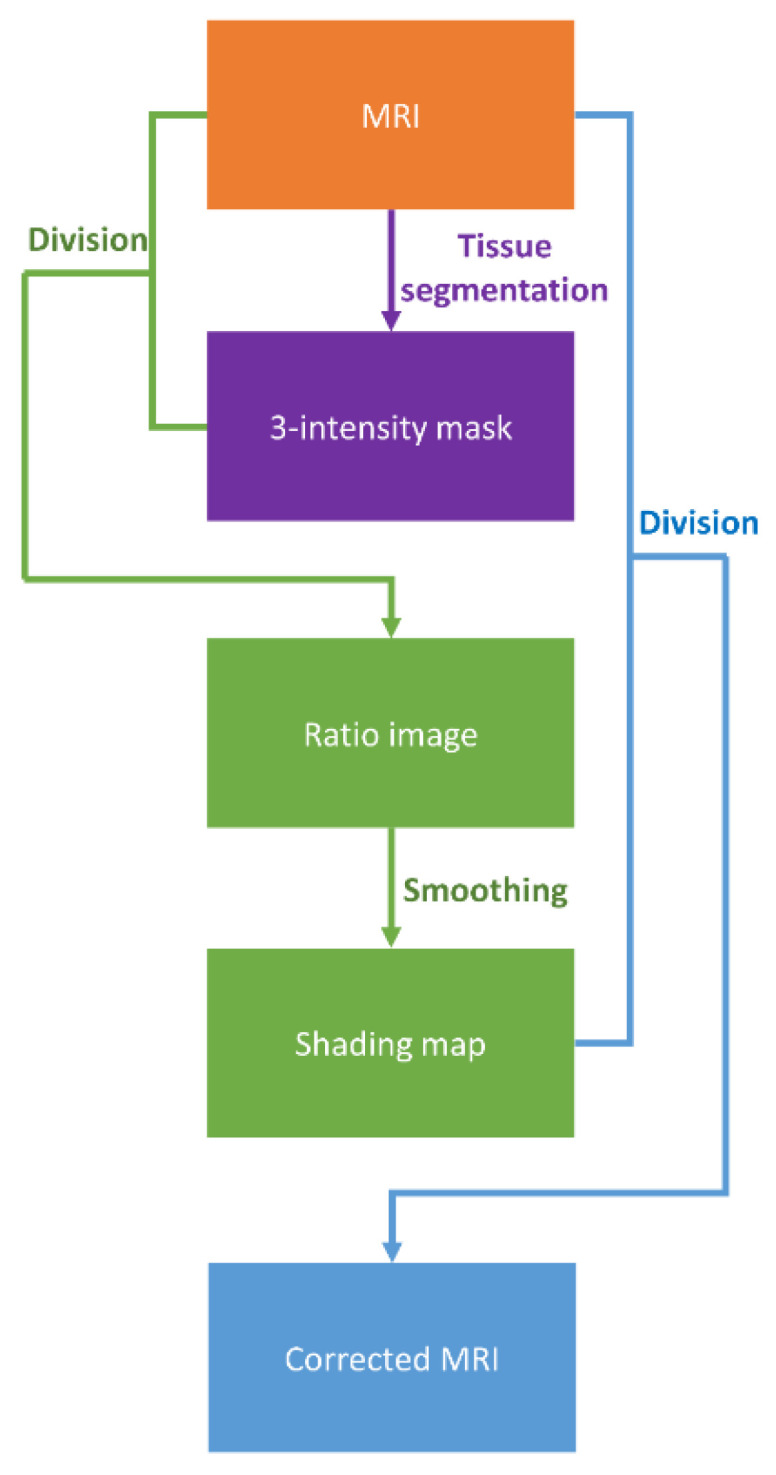
Flowchart illustrating the proposed MRI intensity normalization method.

**Figure 2:**
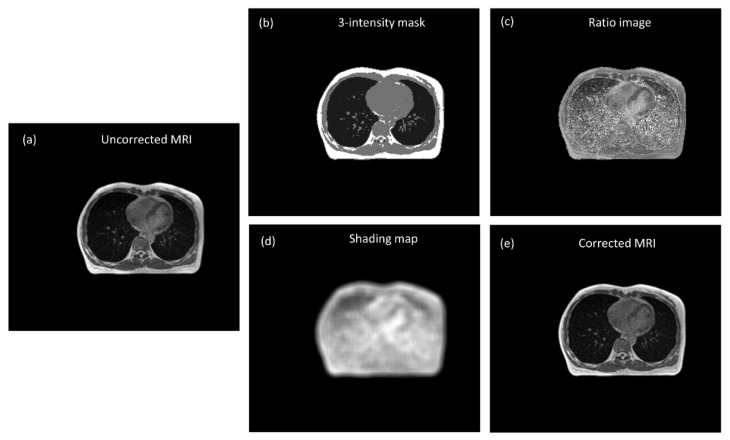
(**a**) Uncorrected MRI (WL/WW = 200/400), (**b**) three-intensity mask, (**c**) ratio image, (**d**) shading map, and (**e**) corrected MRI (WL/WW = 100/200).

Figure 1 illustrates the flowchart of the MR image intensity normalization method proposed in this work, which was adapted from the shading correction method for cone beam CT proposed by Marchant et al. [15] Initially, the MRI was segmented into 3 tissue classes (air/lung, soft tissue, and fat) using the fuzzy c-means (FCM) clustering algorithm [16]. FCM starts with an initial guess for the cluster centers, which moved iteratively by minimizing the distance from any given data point to a cluster center weighted by the fuzzy partition matrix exponent. The mask regions were replaced with a bulk intensity of the corresponding tissue types to generate a three-intensity mask (Figure 2b), whereas the bulk intensity was the mean value from 20 patients receiving the same MRI examination on the 0.35 T system. Next, the MRI was divided by the three-intensity mask to produce a ratio image that indicates the difference between the two images (Figure 2c). The ratio image was then smoothed by an averaging filter with a width of 10 pixels. The smoothed ratio image was referred to as a shading map, which contains intensity inhomogeneity in MRI (Figure 2d). In the last step, the MRI was divided by the shading map for intensity normalization. MATLAB 7.1 (The Mathworks, Natick, MA, USA) was used to perform all image processing steps mentioned above. Since the shading map contains only the slowly varying differences in intensities to be corrected, the high spatial frequency content in MRI can be preserved after correction (Figure 2e).

### 2.4. Pseudo-CT Synthesis from MRI

**Figure 3:**
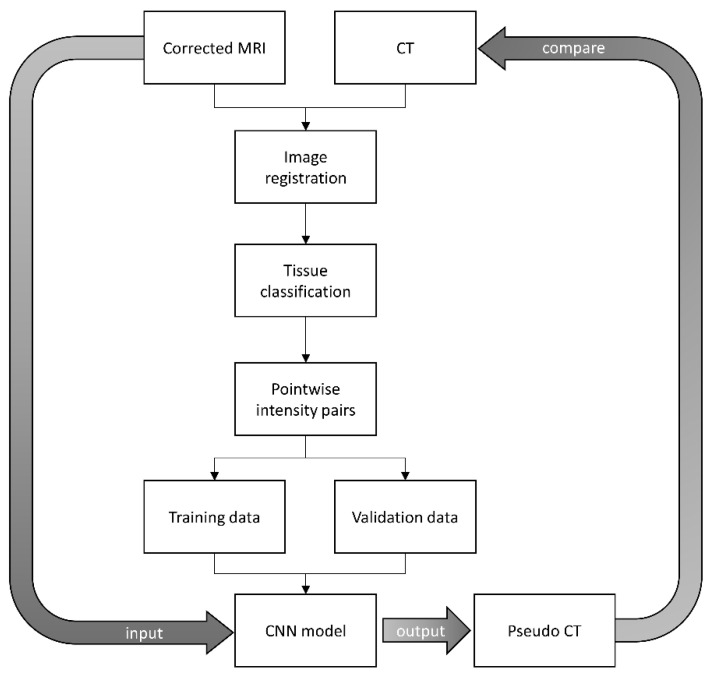
The processing steps of CNN model training and validation to convert corrected MRI into pseudo-CT.

**Figure 4:**
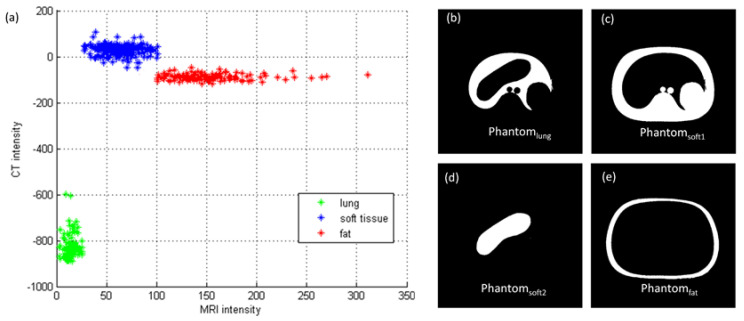
(**a**) Pointwise intensity pairs and phantom image segmented into (**b**) lung, (**c**) low-intensity soft tissue, (**d**) high-intensity soft tissue, and (**e**) fat.

Image registration is the first step of pseudo-CT synthesis, which consists of rigid body registration followed by deformable image registration, whereas in-room MRI was the fixed volume (reference image) and planning CT was the moving volume (deformed image). The similarity measure for the multimodal 3D image registration was based on the Mattes mutual information [17]. The marginal and joint probability density function was evaluated at 50 uniformly spaced bins using 500 samples. Entropy values were computed by summing over the bins. Zero-order and third-order B-spline kernels were used to compute the probability density functions of the fixed and moving images. After registration, the deformed CT images have the same matrix size and voxel size as those in MR images. The registered image pairs were then used in building a convolutional neural network (CNN) model proposed by Nie et al., which converts MRI into pseudo-CT [18]. Theoretically, the registered image pairs should be used as the input (MRI) and label (deformed CT) for CNN model training and validation. However, registration error owing to breath motion, internal organ distortion, and stomach/bladder filling between MRI and deformed CT would severely affect the efficacy of the CNN model. Therefore, instead of using registered MRI-CT image pairs directly for model training and validation, pointwise intensity pairs were extracted from registered image pairs to generate training and validation datasets. Figure 3 illustrates the processing steps of CNN model training and validation to convert corrected MRI into pseudo-CT. After image registration, probabilistic tissue classification was performed by using the FCM clustering algorithm to segment MRI into 3 tissue classes, namely, air/lung, soft tissue, and fat. Voxels within the mask regions were sorted according to their MRI intensities. Next, 25 pointwise intensity pairs evenly covering the MRI intensity range of the mask regions were picked per slice for lung and fat, while 50 pointwise intensity pairs were picked per slice for soft tissue. Only chest imaging for cancer treatment was used to extract pointwise intensity pairs, which were collected from slice numbers 2, 4, 6, and 8 to generate CNN training datasets and slice number 20 to generate validation datasets (Figure 4a). The intensity pairs were fed into 4 phantom templates representing lung (Phantom_lung_, Figure 4b), low-density soft tissue (Phantom_soft1_, Figure 4C), high-density soft tissue (Phantom_soft2_, Figure 4d), and fat (Phantom_fat_, Figure 4e). The input and label images were prepared as 32 × 32-pixel sub-images randomly cropped from the original image. To avoid border effects, all the convolutional layers have no padding, and the network produces an output image with 18*18 matrix size. The training and validation datasets provide roughly 48,862 and 22,825 sub-images, respectively. The CNN model consists of 3 convolutional stages with deeply supervised nets (DSN) to supervise features at each convolutional stage, enabled by layer-wise dense connections in both backbone networks and prediction layers. The model was trained using stochastic gradient descent with mini-batch size of 128, learning rate of 0.01, and momentum of 0.9. The image registration and processing were performed in MATLAB 7.1 on a Windows 10 system (version 2004) with an Intel Core i5-9400 processor. The CNN model was trained and validated by using the Caffe (Convolutional Architecture for Fast Feature Embedding) CNN platform (version 1.0.0-rc5 with CUDA 8.0.61) on an Ubuntu server (version 16.04.4 LTS) with two RTX 2080 (NVIDIA) graphics cards.

### 2.5. Evaluation Study

After CNN training and validation, the model was tested by comparing the pseudo-CT converted from corrected MRI with real CT. To verify the efficacy of the proposed intensity normalization method in facilitating pseudo-CT synthesis, the testing dataset contains both chest imaging for cancer treatment and abdominal imaging for lesion diagnosis. Besides model testing, quantitative analysis was also performed on both MRI and CT before and after processing. With regards to the evaluation of the MRI intensity normalization method, comparison of histograms for MRI before and after correction was conducted. Moreover, the mean value, standard deviation, and coefficient of variation (CV) of uncorrected MRI segmented into 3 different tissue types were compared with those from corrected MRI. The same analysis procedures were also carried out for pseudo-CT versus real CT comparison.

## 3. Results

### 3.1. Chest Imaging for Cancer Treatment

**Figure 5:**
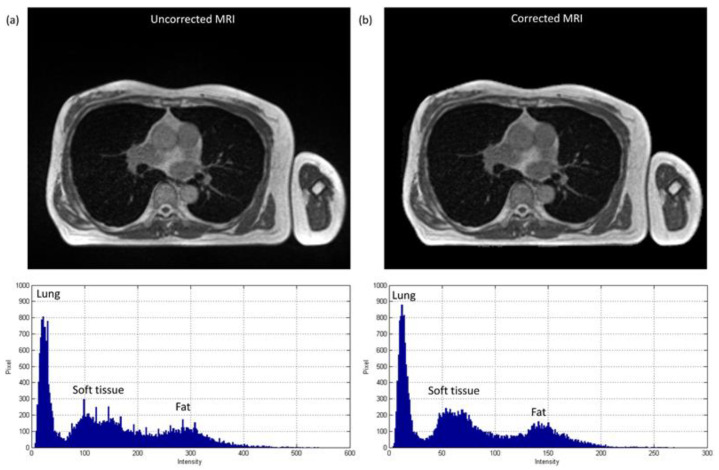
Axial image and histogram of (**a**) uncorrected MRI (WL/WW = 200/400) and (**b**) corrected MRI (WL/WW = 100/200) from 0.35 T scanner for chest imaging used to generate CNN validation dataset (${\mathrm{IMG}}_{\mathrm{validation}}^{\mathrm{chest}}$).

**Figure 6:**
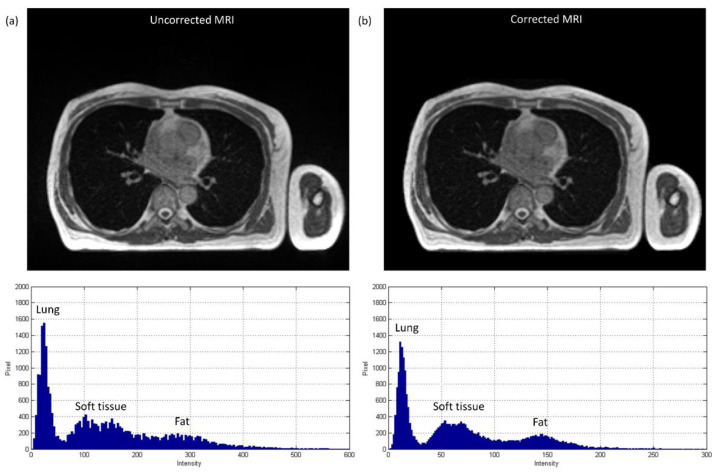
Axial image and histogram of (**a**) uncorrected MRI (WL/WW = 200/400) and (**b**) corrected MRI (WL/WW = 100/200) from 0.35 T scanner for chest imaging divided into CNN testing dataset (${\mathrm{IMG}}_{\mathrm{testing}}^{\mathrm{chest}}$).

**Figure 7:**
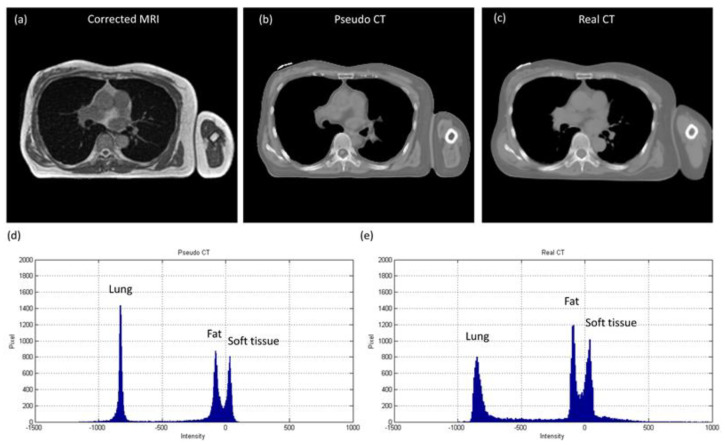
(**a**) Corrected MRI from 0.35 T scanner (WL/WW = 100/200), (**b**) pseudo-CT (WL/WW = 0/1000), (**c**) real CT (WL/WW = 0/1000), (**d**) histogram of pseudo-CT, and (**e**) histogram of real CT for chest imaging used to generate CNN validation dataset (${\mathrm{IMG}}_{\mathrm{validation}}^{\mathrm{chest}}$).

**Figure 8:**
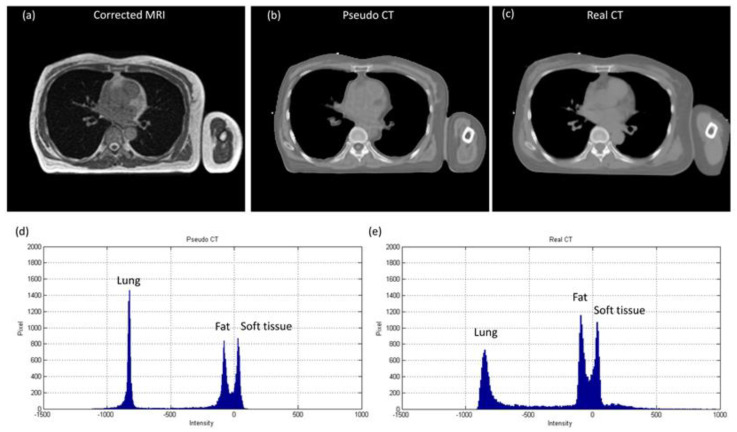
(**a**) Corrected MRI from 0.35 T scanner (WL/WW = 100/200), (**b**) pseudo-CT (WL/WW = 0/1000), (**c**) real CT (WL/WW = 0/1000), (**d**) histogram of pseudo-CT, and (**e**) histogram of real CT for chest imaging divided into CNN testing dataset (${\mathrm{IMG}}_{\mathrm{testing}}^{\mathrm{chest}}$).

**Figure 9:**
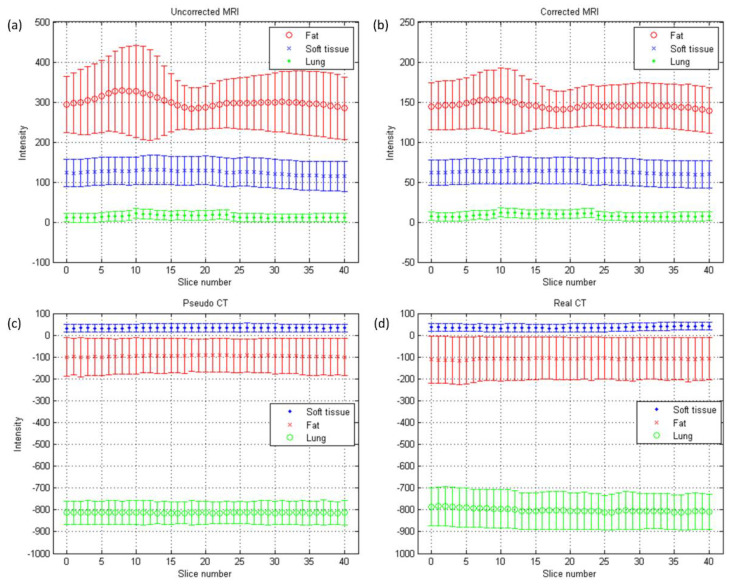
Mean (central line) and standard deviation (error bar) of chest imaging for cancer treatment: (**a**) uncorrected MRI, (**b**) corrected MRI, (**c**) pseudo-CT, and (**d**) real CT.

The axial images and histograms of MRI acquired with the 0.35 T scanner mounted on a LINAC before and after intensity normalization are demonstrated in Figure 5 for chest imaging used to generate the CNN validation dataset (${\mathrm{IMG}}_{\mathrm{validation}}^{\mathrm{chest}}$) and Figure 6 for chest imaging split into the CNN testing dataset (${\mathrm{IMG}}_{\mathrm{testing}}^{\mathrm{chest}}$). For ${\mathrm{IMG}}_{\mathrm{validation}}^{\mathrm{chest}}$, the axial image of corrected MRI acquired with the 0.35 T scanner, its pseudo-CT, and real CT acquired with the 16-slice CT simulator are demonstrated in Figure 7a–c, while the histogram of Figure 7b,c is shown in Figure 7d,e. The corresponding results for ${\mathrm{IMG}}_{\mathrm{testing}}^{\mathrm{chest}}$ are demonstrated in Figure 8. Figure 9 summarizes the mean and standard deviation of image intensity of three different tissue types for uncorrected MRI, corrected MRI, pseudo-CT, and real CT covering 41 slices. As seen in Figure 9, fat tissue is the component showing the most serious MRI intensity inhomogeneity, but its impact on pseudo-CT synthesis was eased after MRI intensity normalization. The mean value ± standard deviation of fat tissue in MRI was 297 ± 73 (CV = 24.58%) before correction and 149 ± 32 (CV = 21.48%) after correction. The mean value ± standard deviation of fat tissue in pseudo-CT was −93 ± 82 HU (CV = 88.17%) and −108 ± 99 HU (CV = 91.67%) in real CT. With regards to soft tissue, the mean value ± standard deviation was 125 ± 35 (CV = 28.00%) in MRI before correction, 64 ± 17 (CV = 26.56%) in MRI after correction, 32 ± 19 HU (CV = 59.38%) in pseudo-CT, and 35 ± 18 HU (CV = 51.43%) in real CT. As for the lung, the mean value ± standard deviation was 26 ± 11 (CV = 42.31%) in MRI before correction, 14 ± 5 (CV = 35.71%) in MRI after correction, −813 ± 53 HU (CV = 6.52%) in pseudo-CT, and −801 ± 84 HU (CV = 10.49%) in real CT. The image slice of ${\mathrm{IMG}}_{\mathrm{testing}}^{\mathrm{chest}}$ demonstrated in Figure 6 and Figure 8 is slice number 14.

### 3.2. Abdominal Imaging for Lesion Detection

**Figure 10:**
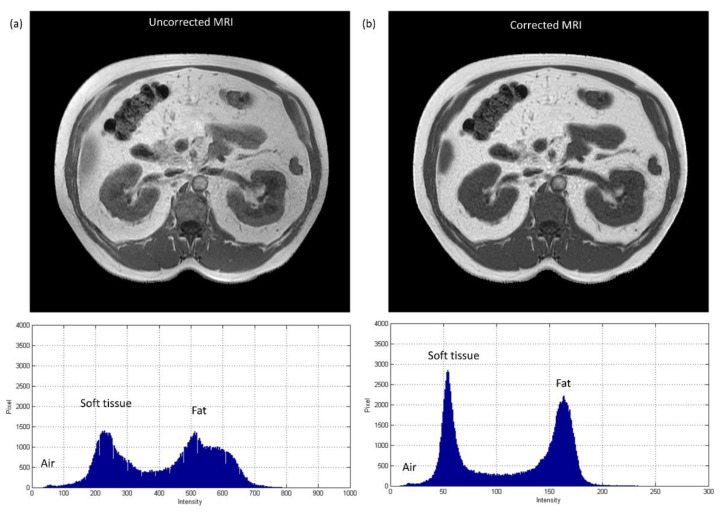
Axial image and histogram of (**a**) uncorrected MRI (WL/WW = 375/750) and (**b**) corrected MRI (WL/WW = 125/250) from 1.5 T scanner for abdominal imaging belonging to CNN testing dataset (${\mathrm{IMG}}_{\mathrm{testing}}^{\mathrm{abdomen}}$).

**Figure 11:**
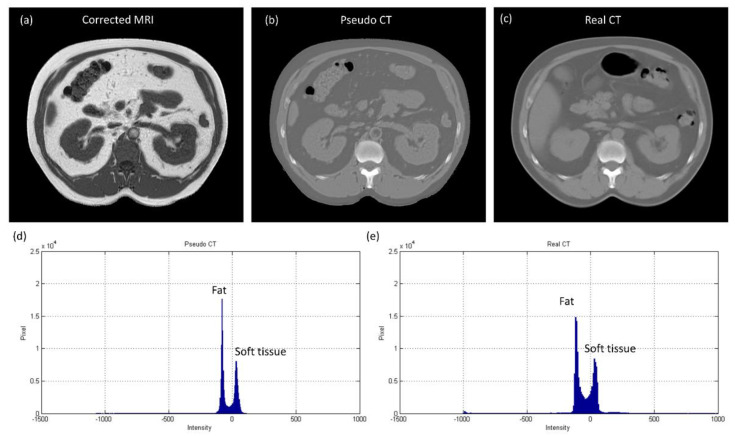
(**a**) Corrected MRI from 1.5 T scanner (WL/WW = 125/250), (**b**) pseudo-CT (WL/WW = 0/1000), (**c**) real CT (WL/WW = 0/1000), (**d**) histogram of pseudo-CT, and (**e**) histogram of real CT for abdominal imaging belonging to CNN testing dataset (${\mathrm{IMG}}_{\mathrm{testing}}^{\mathrm{abdomen}}$).

**Figure 12:**
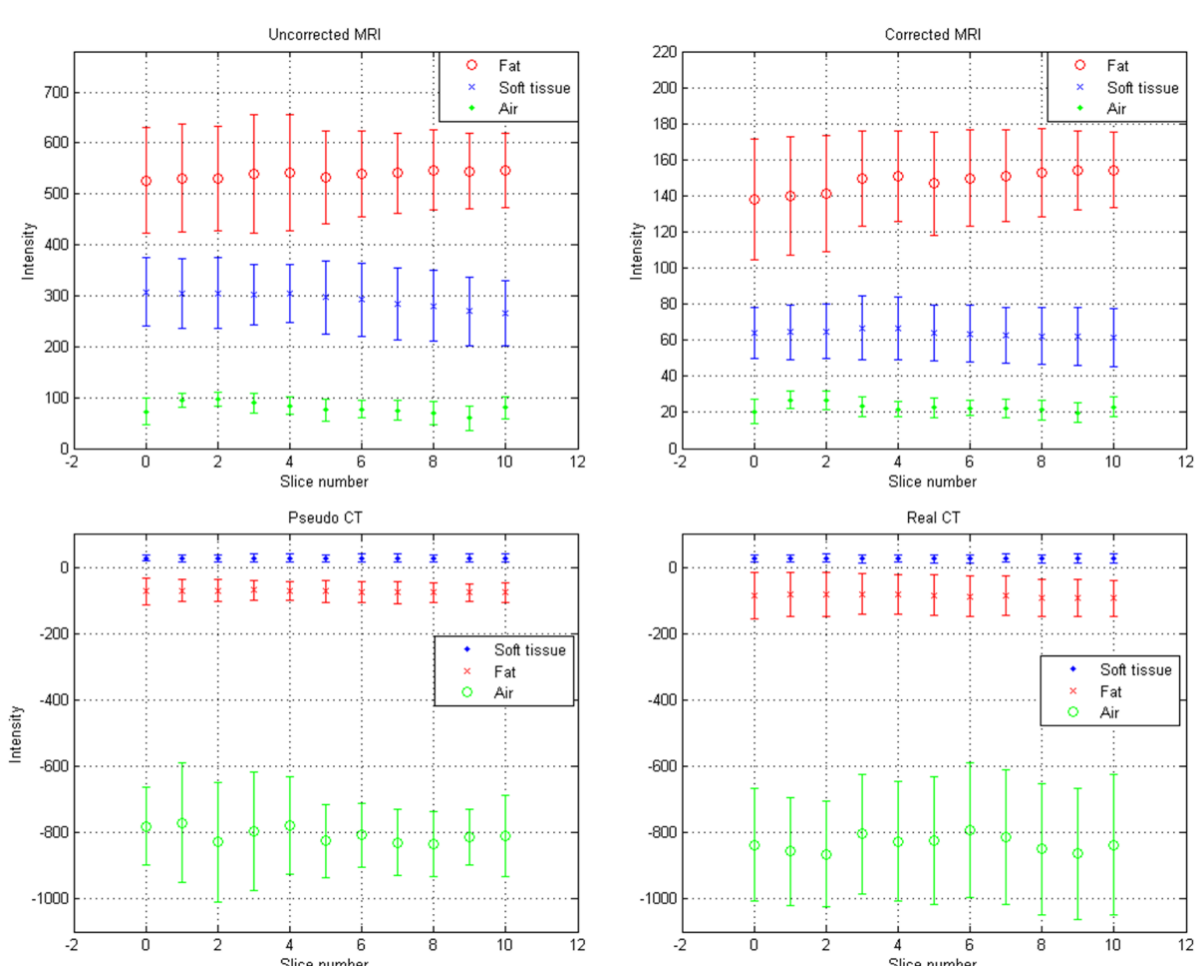
Mean (central marker) and standard deviation (error bar) of abdominal imaging for lesion diagnosis: (**a**) uncorrected MRI, (**b**) corrected MRI, (**c**) pseudo-CT, and (**d**) real CT.

Figure 10 shows the axial images and histograms of MRI acquired with the 1.5 T scanner before and after intensity normalization for abdominal imaging belonging to the CNN testing dataset (${\mathrm{IMG}}_{\mathrm{testing}}^{\mathrm{abdomen}}$). For ${\mathrm{IMG}}_{\mathrm{testing}}^{\mathrm{abdomen}}$, the axial image of corrected MRI acquired with the 1.5 T scanner, its pseudo-CT, and real CT acquired with 64-detector row CT are demonstrated in Figure 11a–c, while the histogram of Figure 11b,c is shown in Figure 11d,e respectively. Figure 12 summarizes the mean and standard deviation of image intensity of three different tissue types for uncorrected MRI, corrected MRI, pseudo-CT, and real CT covering 11 slices. As seen in Figure 12, fat tissue is still the component showing the most serious MRI intensity inhomogeneity, but its impact on pseudo-CT synthesis was eased after MRI intensity normalization. The mean value ± standard deviation of fat tissue in MRI was 533 ± 91 (CV = 17.07%) before correction and 148 ± 28 (CV = 18.92%) after correction. The mean value ± standard deviation of fat tissue in pseudo-CT was −72 ± 32 HU (CV = 44.44%) and −86 ± 63 HU (CV = 73.26%) in real CT. With regards to soft tissue, the mean value ± standard deviation was 298 ± 68 (CV = 22.82%) in MRI before correction, 65 ± 17 (CV = 26.15%) in MRI after correction, 29 ± 11 HU (CV = 37.93%) in pseudo-CT, and 32 ± 15 HU (CV = 46.88%) in real CT. As for air, the mean value ± standard deviation was 65 ± 24 (CV = 36.92%) in MRI before correction, 21 ± 6 (CV = 28.57%) in MRI after correction, −807 ± 129 HU (CV = 15.99%) in pseudo-CT, and −822 ± 127 HU (CV = 15.45%) in real CT. The image slice of ${\mathrm{IMG}}_{\mathrm{testing}}^{\mathrm{abdomen}}$ demonstrated in Figure 10 and Figure 11 is slice number 10.

## 4. Discussion

Since MRI intensities do not have a fixed tissue-specific value, the same tissue could have a wide range of intensities, even within the same protocol, for the same body region, for images of the same subject obtained on the same MRI scanner, which were all found in the MR images acquired with either a 0.35 T or 1.5 T scanner used in this study. Under this physical phenomenon, one of the possible strategies to improve the reliability of MRI to CT conversion is to train and validate the conversion model with datasets covering a variety of imaging conditions, but the size of the datasets may be too huge to be practicable. Hence, this work tried to investigate this issue through another approach, i.e., normalizing MRI intensity of the same tissue type to a similar intensity level. Various intensity normalization methods have been proposed by previous studies, but most of them were focused on brain imaging acquired with 1.5 T MRI scanners [12,14]. Since the torso is the body region investigated in this work and the magnetic strength is only 0.35 T in our MR-LINAC, the resulting artifacts may be different from those presented in previous studies. Hence, an MRI intensity normalization method was proposed and evaluated in the first part of this study. Accurate target delineation is very important in radiotherapy, so one critical characteristic that a proper MRI intensity normalization method for MR-LINAC should have is to preserve the high spatial frequency content in MRI after correction, i.e., edges. Therefore, the initial idea of the proposed intensity normalization method came from the shading correction method used in cone beam CT proposed by Marchant et al. [15]. However, we also found out that our proposed method is a modified version of the method proposed by Axel et al. for correcting intensity inhomogeneity in MRI [19]. Evaluation of the proposed normalization method was performed on chest imaging for cancer treatment and abdominal imaging for lesion detection. At single slice level, it can be found based on naked eye observation and histogram analysis that the inhomogeneity in three different tissue types became more homogenous after correction, especially in fat tissue. Regarding the performance at single scanner level, the variations in mean and standard deviation of MRI segmented into three tissue types of the same body region but at different axial locations were decreased after correction. Moreover, the mean values of 0.35 T corrected MR imaging were similar to those from 1.5 T corrected MR imaging, demonstrating the effectiveness of the proposed method on MRI acquired with different scanners of different body regions. The CVs from uncorrected MRI were similar to those from corrected MRI, demonstrating the preservation of high spatial frequency content.

In pseudo-CT synthesis from MRI, one of the most critical issues that determines the conversion accuracy is the structure consistency between MRI and CT used for model training and validation. Although applying advanced registration methods is one of the possible strategies to deal with this issue, some degree of registration error is still inevitable [20,21]. Another possible strategy is to generate the conversion model based on deep learning methods that could take unpaired MRI and CT, but the computing power and datasets required by unsupervised learning methods limit their clinical practicality [22,23]. Therefore, the CNN training and validation datasets used in this work were generated based on pointwise intensity pairs and phantom templates. The data generation workflow can be realized easily, and the resulting intensity pairs reveal the effective intensity range for MRI to CT conversion. Because the relationship between MRI intensity values and CT numbers can be monitored in our conversion model, accurate conversion was pursued by normalizing MRI intensity into an effective range instead of increasing the amount of datasets for model training and validation. As seen in Figure 7, Figure 8 and Figure 9, the mean values of pseudo-CT segmented into three tissue types were similar to those in real CT, but the intensity ranges in pseudo-CT were slightly narrower than those in real CT. The performance of pseudo-CT synthesized using the conversion model for other patients who underwent 0.35 T scans was similar to the results shown in this work, which is probably due to the similar MRI intensity range (data not shown). To verify the efficacy of our proposed workflow in MRI with an intensity range that is very different from chest imaging for cancer treatment, the conversion model was tested by using abdominal imaging for lesion diagnosis acquired with a 1.5 T scanner. Theoretically, the conversion model that was built based on chest imaging for cancer treatment cannot be applied in abdominal imaging for lesion diagnosis to synthesize pseudo-CT owing to the difference in MRI intensity range between the two datasets (Figure 9a and Figure 12a). Nevertheless, similar intensity ranges were observed in corrected MRI acquired with different systems (Figure 9b and Figure 12b), so the conversion model was also applied to the abdominal MRI acquired with a 1.5 T scanner to evaluate the efficacy of the proposed method. As seen in Figure 11 and Figure 12, the mean values of pseudo-CT segmented into three tissue types were similar to those in real CT, but the intensity ranges were slightly narrower than those in real CT.

Several limitations to this study need to be acknowledged. First, the atlas-based method used in our routine practice was still used to generate pseudo-CT from MRI for bone tissue [3,4]. In radiotherapy, treatment quality is highly correlated with the accuracy and reproducibility of patient positioning. In addition to using immobilization equipment, patient positioning can also be confirmed by locating the bony landmarks. Consequently, the accuracy of registration between MRI and CT is higher in bone than in other tissues. The bone intensities in pseudo-CT shown in Figure 7b, Figure 8b, and Figure 11b are those from real CT after being registered with MRI. There are also several voxel-based methods that were proposed for bone intensity conversion, but most of them require multiple MRI acquisitions or special pulse sequences that cannot be performed on the 0.35 T scanner [11,21,24]. Hence, the atlas-based method was used for bone tissue in this study. Second, the density values in pseudo-CT synthesized via our proposed workflow were compared with those in real CT for two different imaging systems, but no evaluation study was designed to verify the accuracy of radiotherapy dose calculation based on the synthesized pseudo-CT. In chest imaging for cancer treatment, the difference between mean values in pseudo-CT and real CT was 3, 15, and 12 HU for soft tissue, fat, and lung, respectively. As for abdominal imaging for lesion diagnosis, the difference between mean values in pseudo- and real CT was 3, 14, and 15 HU for soft tissue, fat, and air, respectively. These results indicate that the proposed method has substantial reliability in pseudo-CT synthesis for both MRI systems. However, assessment of the proposed workflow in dose calculation for MR-LINAC adaptive plans needs to be further investigated.

## 5. Conclusions

An MRI intensity normalization method was proposed to facilitate pseudo-CT synthesis from MRI. The intra-scanner and inter-scanner reliability of the proposed method were evaluated. Based on our results, fat tissue is the component showing the most serious MRI intensity inhomogeneity. The mean value ± standard deviation of fat tissue in 0.35 T MRI was 297 ± 73, and it was 533 ± 91 in 1.5 T MRI. After MRI intensity normalization, the corresponding results were 149 ± 32 and 148 ± 28. With regards to pseudo-CT synthesis from MRI, the mean values of pseudo-CT segmented into three tissue types were similar to those in real CT, while the intensity ranges were slightly narrower than those in real CT. In chest imaging for cancer treatment, the difference in mean values between pseudo-CT and real CT was 3, 15, and 12 HU for soft tissue, fat, and lung, respectively. The difference in abdominal imaging for lesion diagnosis was 3, 14, and 15 HU for soft tissue, fat, and air, respectively. These results indicate that the proposed method has substantial reliability in pseudo-CT synthesis. Moreover, compared with deep learning methods, which demand a high level of resources for building a conversion model, the workflow presented in this study is more practicable in clinical routine practice.

## Data Availability

The authors confirm that the data supporting the findings of this study are available within the article.
